# BAC array CGH in patients with Velocardiofacial syndrome-like features reveals genomic aberrations on chromosome region 1q21.1

**DOI:** 10.1186/1471-2350-10-144

**Published:** 2009-12-23

**Authors:** Anna Brunet, Lluís Armengol, Damià Heine, Jordi Rosell, Manel García-Aragonés, Elisabeth Gabau, Xavier Estivill, Miriam Guitart

**Affiliations:** 1 Genes and Disease Program, CIBER en Epidemiología y Salud Pública (CIBERESP), Center for Genomic Regulation (CRG), Barcelona, Catalonia, Spain; 2 Genetic laboratory UDIAT-Centre Diagnòstic, Fundació Parc Taulí - Institut Universitari UAB, Corporació Sanitària Parc Taulí, Sabadell, Catalonia, Spain; 3 Hospital Universitari Son Dureta, Mallorca, Spain; 4 Pompeu Fabra University (UPF), Barcelona, Catalonia, Spain

## Abstract

**Background:**

Microdeletion of the chromosome 22q11.2 region is the most common genetic aberration among patients with velocardiofacial syndrome (VCFS) but a subset of subjects do not show alterations of this chromosome region.

**Methods:**

We analyzed 18 patients with VCFS-like features by comparative genomic hybridisation (aCGH) array and performed a face-to-face slide hybridization with two different arrays: a whole genome and a chromosome 22-specific BAC array. Putative rearrangements were confirmed by FISH and MLPA assays.

**Results:**

One patient carried a combination of rearrangements on 1q21.1, consisting in a microduplication of 212 kb and a close microdeletion of 1.15 Mb, previously reported in patients with variable phenotypes, including mental retardation, congenital heart defects (CHD) and schizophrenia. While 326 control samples were negative for both 1q21.1 rearrangements, one of 73 patients carried the same 212-kb microduplication, reciprocal to TAR microdeletion syndrome. Also, we detected four copy number variants (CNVs) inherited from one parent (a 744-kb duplication on 10q11.22; a 160 kb duplication and deletion on 22q11.21 in two cases; and a gain of 140 kb on 22q13.2), not present in control subjects, raising the potential role of these CNVs in the VCFS-like phenotype.

**Conclusions:**

Our results confirmed aCGH as a successful strategy in order to characterize additional submicroscopic aberrations in patients with VCF-like features that fail to show alterations in 22q11.2 region. We report a 212-kb microduplication on 1q21.1, detected in two patients, which may contribute to CHD.

## Background

The hemizygous chromosome 22q11.2 microdeletion occurs in approximately 1:4000-6000 live births [1,2], being the most common genomic aberration among patients clinically diagnosed with velocardiofacial syndrome (VCFS) or DiGeorge syndrome (DGS). Main clinical symptoms include palatal abnormalities, particularly velopharingeal incompetence, with feeding difficulties reported in most (69%) young patients, conotruncal heart defect, characteristic facial features (long face, broad/tubular nose, hooded eyelids, hypertelorism, ear abnormalities and retrognathia), immune deficiency (involving the respiratory tract in up to 77% of cases), motor and developmental delay, learning disabilities, speech delay and language deficits. Behavioural manifestations, such as attention-deficit-hyperactivity disorder (ADHD) and other psychiatric illness in adult patients are also frequent [3-9]. Overall, more than 180 clinical features have been associated with the 22q11.2 microdeletion syndrome http://www.vcfsef.org[9].

The frequency of the 22q11.2 microdeletion varies with the nature of the clinical findings. In infants with a congenital heart defect and no other apparent syndromic features, or with a cleft palate alone, the frequency of chromosome 22q11.2 microdeletion was reported to be very low (0-1% and 1.8%) [10,11]. The wide phenotypic spectrum and the highly diverse clinical features, which frequently overlap with other defined or undefined clinical entities, lead to difficulties in the clinical testing. Other unrelated chromosomal abnormalities have been reported in 2-4% of patients with suspect of VCFS, including microscopic inversions and interchromosomal imbalances, derivatives of parental translocations, marker chromosomes, apparently balanced translocations, ring chromosomes and sex chromosomes aneuploidies [12-15]. Deletions involving contiguous genes on chromosome 10p have also been reported in some patients with VCFS features [16-18], while mutations in gene T-box 1 (*TBX1*) account only for a few of reported cases [19,20].

Nowadays, in genetic testing laboratories, the most common procedure for the diagnosis of this syndrome is by classical cytogenetic karyotyping, which is unable to detect imbalances smaller than 5-10 megabases (Mb), and by fluorescent *in-situ *hybridization (FISH) using commercially available probes N25 or TUPLE1. FISH allows the detection of the most common 3 Mb deletion as well as the nested 1.5 Mb deletion. More recently, PCR-based multiplex ligation-dependent probe amplification (MLPA) has emerged as a cost-effective and accurate diagnostic tool for the analysis of imbalances in the 22q11.2 region. The commercial MLPA assay (Salsa P023B, MRC Holland, The Netherlands) contains probes for several other chromosomal regions such as 10p12-15, 8p21-p23, 4q22-q35, 17p13 and 18q21 based on findings of alterations of these regions in a number of reported patients with DGS/VCFS. However, there is still a subset of patients with VCFS that does not show any chromosomal aberration detectable with the above-mentioned techniques. Many of those VCFS-like patients present common clinical features, like congenital heart disease (CHD), learning disabilities and characteristic facial features, which suggest that a common chromosomal imbalance could be the underlying cause.

Array-based comparative genomic hybridization (aCGH) has successfully been used for detecting genome dosage alterations in children with mental retardation, dysmorphic features and in patients with CHD [21-24]. This technique dramatically increases the resolution and ability to detect copy number alterations compared to conventional cytogenetic methods and allows for a rapid genome-wide screening of submicroscopic copy number aberrations.

Our study was designed to identify submicroscopic genomic alterations important for the pathogenesis of patients with VCFS-like features without the typical 22q11.2 microdeletion. We used two different array designs as screening tools for whole genome and chromosome 22-specific analysis. We describe a novel region on 1q21.1 with a potentially important implication for the CHD and VCFS-like clinical features, and identify several copy number variants (CNVs) inherited from unaffected parents which could contribute to the VCFS-like phenotype.

## Methods

### Patients

A total of 18 patients (9 male and 9 female, aged 5-23 years) with clinical features overlapping the VCF phenotype, referred to our laboratory to rule out 22q11.2 microdeletion, were analysed by custom-made BAC arrays. One sample carrying a 22q11.2 deletion, previously detected by FISH, and 20 normal population samples were also included in the study to control the performance of the arrays. All cases were recruited from the genetics service of the Parc Taulí Health Corporation hospital and had clinical assessments performed by medical doctors with wide experience in treating patients with chromosomal abnormalities. Patients were included in the study on the basis of the presence of VCFS clinical features. Previous laboratory studies on all patients included G-banding karyotype at the level of 600 bands and FISH (Vysis LSI DiGeorge/VCFS region Dual Color Probe) in metaphase and interphase nuclei to rule out the presence of the 22q11.2 microdeletion and microduplication. The presence of CGG-expansions of the *FMR1 *gene and/or subtelomeric abnormalities (MLPA - Salsa P036C and P070, MRC-Holland, Amsterdam, The Netherlands) were also excluded in some patients with mental retardation. Written informed consent was obtained after approval by the ethics board committee of the Parc Taulí Health Corporation.

Genomic DNA was extracted from the whole blood of patients using the Puregene DNA purification kit (QIAGEN-Gentra Systems) according to the manufacturer's protocol. Pooled genomic DNA used as the reference in aCGH experiments was extracted from blood of 50 male or 50 female subjects.

### CGH-BAC arrays

A face-to-face slide hybridization of the sample and reference DNAs on two different arrays was performed (see below). The 5.6 K whole genome BAC array consists of 5,442 large insert DNA fragments (BACs) with a global coverage of 23% of the euchromatic genome and a much higher density in hotspot candidate regions, such as those located between segmental duplications and all subtelomeres. The clone set used to produce this array was mainly derived from the 32 K human BAC library from the Children Hospital Oakland Research Institute http://bacpac.chori.org/home.htm and several gaps in the library were covered using BACs from other libraries (mainly RP11). The distribution of BACs in the array is not homogeneous, but the average spacing between consecutive clones is 0.5 Mb and the maximum 1.2 Mb. The chromosome-22-specific tiling path array consists of 363 genomic BAC clones derived from the human chromosome 22. Slides contained triplicates of all clones providing an average density of at least one clone per 46 kb along the entire euchromatic region of chromosome 22.

The production of the arrays was performed as described by Cuscó et al. (2008) [25], probe preparation and hybridizations were performed in the Microarray Unit of the CRG, Barcelona, Spain. Briefly, for array preparation, BAC DNA was isolated from 1.5 ml bacterial cultures using the Montage BAC96 Miniprep kit following manufacturer's instructions (Millipore, Billerica, MA). DNA amplification by DOP-PCR was done as described in Fiegler et al. (2003) [26]. PCR products were purified using the Montage PCR96 Plates kit (Millipore, Billerica, MA) and quantified using the PicoGreen dsDNA Quantification kit (Invitrogen, Life technologies, Carlsbad, CA). Purified products were dried, dissolved at 400 ng/μl in 50% DMSO and spotted using a VersArray ChipWriter™ Pro System (Bio-Rad).

Arrays were scanned using an Agilent G2565BA Microarray Scanner System (Agilent Inc., Palo Alto, CA) and the acquired images were analyzed using GenePix Pro 6.0 software (Axon, Molecular Devices) using the irregular feature finding option. Extracted raw data was filtered and Lowess normalized using Bacanal (Lozano et al., unpublished), an in house web server implementation of the Limma package developed within the Bioconductor project in the R statistical programming environment. SD of all 22-chromosome clones was calculated for each hybridization experiment. Genomic imbalances were determined based on the log2 of the Cy5/Cy3 ratios of the average of the four replicates and regions were considered as amplified or deleted when at least the absolute value of two consecutive clones exceeded the 0.2 threshold.

### Face-to-face slide hybridization

In order to save time, reagents and reduce experimental noise, the same sample was simultaneously hybridized onto the two different slides by arranging the two arrays face to face in the same hybridization chamber (Corning^® ^Hybridization Chamber II with Increased Depth, Cultek). Reversed-dye labelling of the samples was always performed to minimize the effect of dye bias specific artifacts. Each patient was hybridized against a sex-matched pool of 50 healthy controls. Hybridization was performed as described by Wang et al. (2004) [27]. Briefly, 400 ng of test and reference DNA were labelled by random priming using the BioPrime Array CGH Genomic Labelling System (Invitrogen, Life technologies, Carlsbad, CA). Cy3 and Cy5 labelled test and control DNA precipitate together with 100 μg of human Cot1 DNA and resuspended in 80 yl of hybridization buffer (50% formamida, 2 × SSC, 10% dextran sulphate, 1× denhard's solution, 0.5 mM EDTA pH 8, 40 mM NaPhosphatase pH 7). The two slides which make up the pair were placed facing together slightly offset to create a lip along one edge. The barcodes on the arrays created a small space between the slides. The hybridization solution was slowly and carefully applied along the lip and evenly occupied the space between the slides.

### Validation experiments

The copy number aberrations identified by aCGH, which we considered for further validation, were analysed using other molecular techniques such as MLPA and FISH analysis. When parental samples were available we checked by MLPA the inheritance pattern of the alterations. The MLPA probes were developed according to the procedures described elsewhere http://www.mrc-holland.com and using the MLPA Proseek algorithm [28]. The MLPA reactions were performed as described by Schouten et al., 2002 [29]. For the data analysis we calculated the relative probe signals using the peak heights of PCR products. Briefly, the tracing data was normalized by dividing each probe's peak height by the total height of all peaks of the sample and then dividing this value by the average normalized peak's height of the corresponding locus of all the samples. The product of this calculation is termed dosage quotient (DQ). A DQ value below 0.65 was considered as indicative of a deletion, and values above 1.3 are indicative of duplications.

To confirm array results in patient V5, we performed fluorescence in situ hybridization (FISH) experiments following standard procedures. Furthermore to better characterize the 1q21.1 microdeletion and microduplication of the 1q21.1 region in both cases (V5 and 112) we used a custom array consisting of 130,000 isothermal probes with 1,155 probes in the 1q21.1 region based on Build 36 coordinates (chr1:143,500,000-148,000,000). The experiments and the subsequent analyses of aCGH were performed as previously described in detail [25,30].

## Results

The whole-genome 5.6 K and the chromosome 22-specific BAC-arrays were used to study 18 patients with the VCFS-like phenotype. We identified a total of 81 clones with genomic dosage changes, belonging to 45 different chromosomal regions (14 losses, 14 gains and 17 with signals in both directions) [see Additional file 1: Supplemental Table S1]. All chromosomal changes were compared with the information deposited in the Database of Genomic Variants (Build 36) http://projects.tcag.ca/variation/[31]. 39 loci had been reported as CNVs in the database and 20 of them were also detected as common variants in 20 control samples previously hybridized on the same 5.6 K BAC-array [25]. The aim of this study was screening for submicroscopic deletions and duplications underlying several malformations such as heart defect, palatal abnormalities or mental delay, for this reason individual clones that were reported polymorphic in normal individuals and also the loci identified as variable in our control samples were excluded. We selected eleven loci for further MLPA validation, none of which were present in control samples: six had not been previously described as CNVs and five regions overlapped with previously reported CNVs, but had been chosen after the imbalance of various consecutive clones in the samples (encompassing regions >150 kb and up to 1.15 Mb) [see Additional file 1: Supplemental Table S1]. Five of the eleven CNVs identified by CGH were validated by MLPA and screened by the same technique in parental samples (Table 1). The remaining six loci could not be validated by MLPA although at least two different probes per region were assayed. Oligonucleotide sequences used for MLPA are shown in Additional file 1: Supplemental Table S2.

**Table 1:** Summary of altered regions, parental analysis and major clinical features in patients with velocardiofacial-like syndrome (VCFS-like) validated by multiplex ligation-dependent probe amplification (MLPA).

Sample GAIN	Sample LOSS	Altered clones	Region	Size kb	Start (Build 36)	End (Build 36)	Genes	LOCUS (DGV)	MLPA probes	Inheritance	Clinical features
V5		RP11-315I20 RP11-293J20	1q21.1	212	144149999	144361868	TXNIP, POLR3GL, ANKRD34, LIX1L, RBM8A, PEX11B, ITGA10, ANKRD35, PIAS3, NUDT17, POLR3C, ZNF364	Locus 0305	GNRH2 PIAS3	*de novo*	Cardiac anomalies (coarctation of the aorta); Velopharyngeal insufficiency; dysmorphic facial features; language impairment, mental retardation, cognitive and learning problems, immunological abnormalities.
	V5	RP11-337C18 RP11-533N14 RP11-314N2 RP11-301M17 RP11-115G11	1q21.1	1,150	145073765	146329018	PRKAB2, FMO5, CHD1L, BCL9, ACP6, GJA5, GJA8, GPRB9	Locus 0305	BCL9 NBPF1	*de novo*	
V26		RP11-314P12 RP11-292F22 RP11-192A16 RP11-30N1 RP11-115A11	10q11.22	743,52	46487806	47231326	PPYR1, ANXA8L, CTGLF4	Locus 2984	PPYR1	Paternal	Mild dysmorphic facial features, mild mental retardation, psychological anomalies, phobias
V8	V24	RP11-818K20 RP11-444L7	22q11.21	159	19847992	20006849	-	Locus 4746	RP11-444L7	Both Paternal	V8: Dysmorphic facial features, learning disabilities mental retardation, father with schizophrenia V24: Overt submucos palate, mild facial dysmorphic features, mild mental retardation, learning disabilities, autistic behaviour, (brother with cardiac anomalie)
V11		RP11-138G4	22q13.2	142	39422672	39564831	SLC25A17, ST13		SLC25A17b	Maternal	Dysmorphic facial features, learning disabilities, language impairment, attention deficit disorder

DGV, Database of Genomic Variants.

### Characterization of *de novo *genomic imbalances on chromosome 1q21.1

**Figure 1:**
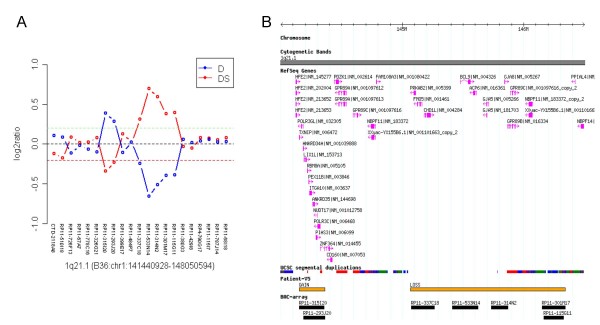
**Genomic imbalances on chromosome 1q21.1**. **A: **5.6K whole genome BAC aCGH profiles for patient V5 on chromosome region 1q21.1. Each dot represents the mean log2 ratio transformed after Lowess normalization (y-axis) from four independent replicate spots on the array. The clones on the 1q21.1 region are displayed in the x-axis. Direct experiments (D) are shown in blue, while dye swap (DS) experiments are displayed in red. Each dot represents a BAC clone present in the aCGH experiment. **B: **Genome browser representation of the 1q21.1 region containing the microduplication/microdeletion. The figure shows the related segmental duplications that probably mediated the rearrangements and the genes affected. The microduplication of 212 kb (reciprocal to the TAR syndrome micodeletion), and the downstream microdeletion of 1.15 Mb are indicated by two orange bars (GAIN and LOSS).

**Figure 2:**
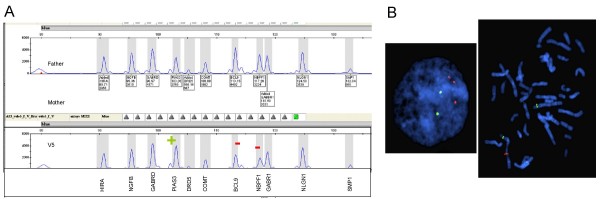
**Detection and validation of the chromosome 1q21.1 rearrangements in patient V5**. **A: **Electropherograms showing MLPA peak patterns corresponding to familial analysis of case V5. **B: **Interphase and metaphase FISH images with 1q21.1 red BAC probes confirm the microduplication (RP11-293J20) and microdeletion (RP11-314N2). A Green probe generated from CTD-2180H16 BAC clone was used as a control probe from same chromosome (1p34.2).

An interstitial microduplication spanning two overlapping BAC clones, with an estimate size of 212 kb, and a microdeletion spanning five consecutive clones, with an estimate size of 1,15 Mb, on region 1q21.1 were found to occur *de-novo *in patient V5. The duplicated region (aCGH 1q21.1(B36:chr1:144149999-144361868++)) included 12 RefSeq Genes (*TXNIP, POLR3GL, ANKRD34A, LIX1L, RBM8A, PEX11B, ITGA10, ANKRD35, PIAS3, NUDT17, POLR3C, ZNF364*), and the deleted region (aCGH 1q21.1 (B36:chr1:145073765-146329018)) encompassed eight genes (*PRKAB2, FMO5, CHD1L, BCL9, ACP6, GJA5, GJA8, GPR89B, GPR89C, NBPF11*) (Figure 1). FISH and MLPA analysis confirmed both rearrangements in patient V5, and excluded their presence in his parents (Figure 2).

**Figure 3:**
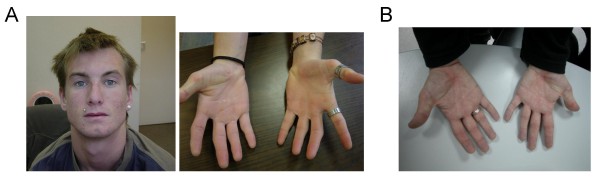
**Facial appearance and hands of patients with 1q21.1 rearangements**. **A: **Patient V5 with microduplication/microdeletion 1q21.1: note frontal balding, arched eyebrows, deep-set eyes and thin upper lip, fingers with camptodactyly and mild interdigital membranes. **B: **Patient 112 with microduplication 1q21.1: Note moderate clinodactyly.

This patient is a 17 year old male with cardiac anomalies (coarctation of the aorta), some dysmorphic facial features (frontal balding, arched eyebrows, eyes deeply set and thin upper lip), velopharyngeal insufficiency with reported feeding problems in infancy (gastroesophageal reflux and frequent vomiting), language impairment, moderate mental retardation, low cognitive deficit and learning problems (irritability, attention deficit hyperactivity disorder), immunological abnormalities (frequent bronchitis), asthma, strabismus (surgery corrected at two years), small umbilical hernia, and fingers with mild camptodactyly, mild interdigital membrane and flat foot arches (Figure 3-A). This patient was included in a previously published series that reported 22 probands with the 1q21.1 microdeletion [30]. We provide here additional clinical information of this case.

We screen the 1q21.1 region, on 73 additional cases with congenital heart defects by MLPA. We found one patient (case 112) with the same microduplication but without the contiguous deletion on 1q21.1, also present in his unaffected father. The clinical features of this patient were cardiac abnormalities (transposition of the great vessels, ventricular septal defect, pulmonary stenosis and right ventricular hypoplasia), braquicephaly and moderate clinodactyly (Figure 3-B). We also analysed 326 additional control DNA samples obtained from anonymous unrelated blood donors using the same MLPA probe mix. All control samples were negative for both rearrangements.

To carefully delineate alterations in the 1q21.1 region, the samples V5 and 112 were also hybridized with a custom oligo array with 1,155 probes being from the 1q21.1 region, as described in [30]. Results confirmed the complex microduplication/microdeletion in case V5 and that both patients shared the same microduplication, reciprocal to the TAR syndrome microdeletion [see Additional file 2: Supplemental Figure S4].

### Identification of inherited aberrations

Four of the validated aberrations were CNVs inherited from one of the parents (Table 1), three overlapping with loci previously reported as CNVs in the reference Database of Genomic Variants http://projects.tcag.ca/variation/[31]. A 744-kb duplication on 10q11.22 involving five clones was identified in patient V26, showing facial dysmorphism, learning disability, mild mental retardation and behavioural problems. MLPA with one specific probe on *PPYR1 *showed a gain in the patient and his father.

With the chromosome 22 tiling array we identified aberrations involving the deletion of two clones on the 22q11.2 region (160 kb) in two cases. Patients V8 and V24 with dysmorphic facial features, low mental retardation and behavioural problems showed a gain and a loss respectively, both paternally inherited. Finally, a gain of BAC RP11-138G4, located on 22q13.2, which was maternally inherited, was also identified with this chromosome-specific array. This region has not been reported in the literature as a CNV and spans the *SLC25A17 *gene that encodes a peroxisomal membrane protein belonging to the family of mitochondrial solute carriers [OMIM *606795]. This BAC clone also includes six exons of the *ST13 *gene, encoding a highly conserved protein that binds the major cytosolic chaperones heat-shock proteins HSP70 and HSP90.

## Discussion

The VCFS is a genomic disorder due to a hemizygous deletion on chromosome 22q11.2. Our set of 18 patients showed clinical features compatible with VCFS- like cardiac anomalies, learning disabilities and characteristic facial features but lacked the typical 22q11.2 deletion. We screened our patients with VCF-like phenotypes with two different custom-made aCGH (BAC-based whole genome and chromosome 22 specific tiling path). The genome-wide analysis of structural variations allowed us to identify *de novo *genomic rearrangements and inherited CNVs in these patients.

Five out of the 11 altered regions selected for validation were confirmed by MLPA, while the other six showed no variation. Although the non-confirmed CNVs were screened with two MLPA different probes, indicating that they could be aCGH false positives, we have to consider the possibility that the designed MLPA probes might lie outside the BAC variable region. Recent reports indicate that the size of the CNVs identified by BAC arrays is likely to be overestimated. In fact, the concordance rate for CNVs identified by two different platforms (BAC vs. SNP arrays) was less than half (43%) when studying the same individuals [32]. Concerning the five validated variations, the analysis of parental samples revealed that four of them were inherited. The presence of parents carrying these CNVs but without clinically evident phenotypes raises the issue of whether these CNVs are benign or pathogenic variants with incomplete penetrance. Indeed, some well-characterized syndromes such as the 22q11.2 microdeletion or the reciprocal microduplication, include phenotypically mild deletion carriers that have escaped clinical recognition until they had children with more severe manifestations. Furthermore, it is still possible that the presence of an inherited CNV, especially deletions, could be uncovering a recessive allele inherited from the other parent. Further work in this direction would be required to evaluate these hypotheses.

Two *de-novo *chromosome rearrangements were identified by aCGH and MLPA analysis in one patient with VCFS-like features. This is, to our knowledge, the first time that this combination of two CNVs (an interstitial microduplication, spanning 2 BAC clones with an estimate size of 212 kb (chr1: 144149999-144361868) and a contiguous microdeletion spanning 5 BAC clones with an estimate size of 1.15 Mb (chr1:145068638-146342725) is found associated with such a phenotype.

This 1.15-Mb microdeletion was first reported in three cases of CHD. The authors screened by real-time quantitative PCR a total of 505 unrelated congenital heart disease cases for deletions or duplications of Cx40 gene (*GJA5*) and identified three cases with a 1.5 to 3-Mb deletion of this region; however the microdeletion was also present in some unaffected parents [33]. Recently, this 1.15 Mb microdeletion has been associated with schizophrenia and it has also been reported in individuals with considerable phenotypic diversity including cardiac abnormalities, mild-to-moderate mental retardation, microcephaly, cataracts, mullerian aplasia, and autism [30,32-39]. Nowadays, the main clinical features associated with this rearrangement are still unclear and the evaluation of family members has revealed apparently unaffected carriers, making genetic counselling difficult. However, the absence of this rearrangement in more than 5,000 normal individuals analyzed suggests that this structural variant is rare, and probably contains important modifiers since it exhibits incomplete penetrance.

Eight genes of this region could be involved in a wide variety of phenotypic features, at least two potentially involved in cardiac defects: *PRKAB2 *and *GJA5 *(Cx40), both expressed in the heart. A *Cx40 *heterozygous knockout mice (+/-) has been shown to develop a number of cardiac malformations including bifid atrial appendage, ventricular septal defect, tetralogy of Fallot and aortic arch abnormalities [40]. *GJA8 *has previously been reported associated with schizophrenia [41].

Although previous individuals have been found to carry this microdeletion (1.15 Mb), this is the first time that it is reported in concurrence with the contiguous microduplication (212 kb). The only evident clinical feature shared by the two cases reported here is the CHD. This region contains numerous segmental duplications that could mediate genomic rearrangements. Some larger rearrangements encompassing the 1q21.1 region have been reported associated with varying phenotypes that included dysmorphic features, hypotonia or mental retardation. Among those, there are some cases with supernumerary marker or ring chromosomes derived from the pericentromeric region of chromosome 1 plus q-arm euchromatic fragments, [42-45]. Furthermore, it has been reported that the reciprocal microdeletion of 1q21.1 is necessary but not sufficient to cause TAR syndrome [46], characterized by bilateral absence of the radii and thrombocytopenia, also the lower limbs, gastrointestinal, cardiovascular and other systems may be involved [47].

## Conclusions

In summary, this is the first study in which aCGH is used to investigate patients with VCFS-like phenotype without the classical 22q11.2 molecular rearrangement. aCGH provides a successful strategy in order to characterize additional submicroscopic aberrations in patients that fail to show alterations in 22q11.2. We have identified a rare and *de novo *1q21.1 microdeletion and a novel microduplication that could be associated with CHD. Further new cases shall contribute to delineate more precisely the clinical implications of these newly recognized genomic alterations on 1q21.1 loci and their implication in CHD.

## List of abbreviations used

aCGH: Array-based comparative genomic hybridization; ADHD: attention-deficit-hyperactivity disorder; CHD: congenital heart defects; CNVs: Copy number variants; DGS: DiGeorge Syndrome; FISH: fluorescence *in situ *hybridization; MLPA: multiplex ligation-dependent probe amplification; TAR: thrombocytopenia-absent radius; VCFS: Velocardiofacial syndrome.

## Competing interests

The authors declare that they have no competing interests.

## Authors' contributions

AB participated in the construction and validation of the tiling-path 22 chromosome CGH array, analysis and interpretation of array results, carried out the FISH and MLPA analysis and drafted the manuscript. LLA participated in the design of the study, construction and validation of the whole genome array, interpretation of data and helped to draft the manuscript. DH contributed with samples collection of patients with CHD and carried out of MLPA analysis on 1q21.1 region. JR contributed with clinical evaluation of patient 112 and revision of clinical data. MG-Aparticipated in the design, construction and validation of the whole genome and tiling-path 22 chromosome CGH array, analysis and interpretation of array results. EG contributed with clinical evaluation of VCFS-like patients, samples collection of their parents and participated in the revision of clinical data reported in the manuscript. XE and MG participated in the conception, design and coordination of the study, in the revision of the manuscript and final approval of the version. All the authors read and approved the final manuscript.

## Pre-publication history

The pre-publication history for this paper can be accessed here:

http://www.biomedcentral.com/1471-2350/10/144/prepub

## Supplementary Material

Additional file 1 **Supplementary Table S1 - S3**. **Table S1: **The table provide the copy number changes detected by BAC array-CGH. Comparison of the presence or absence of 44 CNV regions in patients with velocardiofacial-like syndrome (VCFS-like) and control samples. Numbers indicate gains or losses of each BAC. The eleven regions validates by MLPA are highlighted. **Table S2: **Selected regions for validation studies and oligonucleotide sequences used for detecting copy number changes by MLPA in patients with VCFS-like. **Table S3: **Selected oligonucleotide sequences used for detecting CNVs on 1q21.2 region by MLPA in patients with VCFS-like. Click here for file

Additional file 2 **Supplementary Figure S4**. Array CGH results for deletion cases V5 and 112 (Build 36, chr1:143,500,000-148,000,000). For each patient, deviations from 0 of probe log_2 _ratios are depicted by vertical bars, with those exceeding a threshold of 1.5 SD from the mean probe ratio shown in green (gains) or red (losses). The region of the microdeletion associated with TAR syndrome is indicated with a circle. Click here for file
