# Supplementary material for: BAC array CGH in patients with Velocardiofacial syndrome-like features reveals genomic aberrations on chromosome region 1q21.1
Source: BMC Med Genet. 2009 Dec 23;10:144. doi: 10.1186/1471-2350-10-144 (PMC2805625; doi:10.1186/1471-2350-10-144)
Supplement: Additional file 1 — Supplementary Table S1 - S3. Table S1: The table provide the copy number changes detected by BAC array-CGH. Comparison of the presence or absence of 44 CNV regions in patients with velocardiofacial-like syndrome (VCFS-like) and control samples. Numbers indicate gains or losses of each BAC. The eleven regions validates by MLPA are highlighted. Table S2: Selected regions for validation studies and oligonucleotide sequences used for detecting copy number changes by MLPA in patients with VCFS-like. Table S3: Selected oligonucleotide sequences used for detecting CNVs on 1q21.2 region by MLPA in patients with VCFS-like. [file 1471-2350-10-144-S1.DOC]

**Supplementary Table S1**

| **Chr** | **Region** | **Size (kb)** | **CASES** | | **CONTROLS** | | **Altered clones** | **Start** | **End** | **LOCUS (Database of Genomic Variants)** | **Affected genes** | **Validate by MLPA** |
| --- | --- | --- | --- | --- | --- | --- | --- | --- | --- | --- | --- | --- |
|  |  |  | **GAIN** | **LOSS** | **GAIN** | **LOSS** |  |  |  |  |  |  |
| 1 | 1p36.33 | 0 |  | 1 |  |  | RP11-772F06 |  |  | Locus 0052 Locus 0053 | PUSL1, CPSF3L, TAS1R3, DVL1, MXRA8, MRPL20, WARP, FLJ37183, ATAD33 |  |
| 1 | 1p36.21 | 660 |  | 1;1 |  | 1;1 | RP11-583G23 RP11-707I5 | 12730060 | 13389999 | Locus 0130 | C1orf158, DHRS3, PRAMEF3, PRAMEF4, HNRPCL1, PRAMEF2, PRAMEF9, PRAMEF7, PRAMEF8, RP11-474O21.1, PRAMEF5, PRAMEF6, PRAMEF1, PRAMEF15, PRAMEF14, PRAMEF18, PRAMEF19, PRAMEF16 |  |
| 1 | 1p36.11 | 180 |  | 4 |  |  | RP11-335G20 | 25327725 | 25507851 | Locus 0157 | RHD, TMEM50A, RHCE |  |
| 1 | 1p21.3 | 162 | 1 |  |  |  | RP11-29O12 | 96478367 | 96640177 | NO CNV |  | NO |
| 1 | 1p21.1ishClones | 119 | 8 | 2 |  |  | RP5-1108M17 | 103893839 | 104012549 | Locus 0273 | AMY2B, AMY2A |  |
| 1 | 1q12 | 103 |  | 2 |  |  | CTD-2118A8 | 120568788 | 120671324 | Locus 0299 | FAM72B |  |
| 1 | 1q21.1 | 212 | 1;1 |  |  |  | RP11-315I20 RP11-293J20 | 144149999 | 144361868 | Locus 0305 | HFE2,TXNIP,POLR3GL,ANKRD34A,LIX1L,RBM8A, PEX11B,ITGA10, ANKRD35, PIAS3, NUDT17, POLR3C, ZNF3G4 | YES |
| 1 | 1q21.1 | 1148 |  | 1;1;1;1;1 |  |  | RP11-337C18 RP11-533N14 RP11-314N2 RP11-301M17 RP11-115G11 | 145073765 | 146329018 | Locus 0305 | PRKAB2, FM05,CHD1L, BCL9, ACP6, GJA8, GPR89A, NBPF1, NBPF1, FLJ39739 | YES |
| 1 | 1q21.1 | 172 |  | 2 |  |  | RP11-429I8 | 147845219 | 148016956 | Locus 0305 |  |  |
| 2 | 2p22.1 | 179 |  | 1 |  |  | RP11-457F14 | 40346699 | 40526041 | Locus 0550 | SLC8A1 |  |
| 2 | 2p11.2 | 88 | 1 |  |  | 1 | CTD-2148K13 | 87512069 | 87600376 | Locus 0641 |  |  |
| 2 | 2p11.2 | 211 |  | 1 | 2 | 1 | RP11-316G9 | 89561552 | 89772752 | Locus 0643 |  |  |
| 2 | 2q13 | 165 | 1 |  | 2 | 1 | RP11-330B16 | 112061368 | 112226421 | Locus 0672 |  |  |
| 2 | 2q21.1 | 165 | 1 |  |  |  | RP11-32C20 | 130418003 | 130583445 | Locus 0692 | RAB6C, A26C1B |  |
| 5 | 5q13.2 | 61 |  | 3;1 | 2;1 | 3;0 | RP11-551B22 RP11-195E2 | 70362440 | 70423291 | Locus 1723 | DKFZ686M0199, LOC728340, GTF2H2, LOC730394 |  |
| 5 | 5q35.1 | 171 |  | 1 |  |  | RP11-292M11 | 169611921 | 169783365 | NO CNV | LCP2, KCNIP1, KCNMB1 | NO |
| 6 | 6p25.3 | 179 | 1 | 3 |  |  | RP11-328C17 | 177604 | 356249 | Locus 1887 | DUSP22, IRF4 |  |
| 6 | 6p26 | 198 | 1;0 | 4;3 | 4;2 | 2;0 | CTD-2310B5 RP11-713A24 | 160912548 | 161110580 | Locus 2178 | ANXA4, LPA, PLG |  |
| 8 | 8p23.1 | 120 | 2 |  |  | 1 | RP11-743F19 | 7138613 | 7258467 | Locus 2456 |  |  |
| 8 | 8p23.1 | 717 | 1;3 | 3;3 | 1;1 | 2;2 | RP11-115E11 RP11-739E3 | 7176292 | 7893166 | Locus 2456 | DEFB103A, DEFB103B, SPAG11B, DEFB104A, DEFB104B, DEFB106A, DEFB106B, DEFB105A, DEFB105B, DEFB107A, DEFB107B, SPAG11A, DEFB103A, DEFB103B, DEFB4 |  |
| 8 | 8p12 | 177 |  | 1 |  | 3 | RP1-144M5 | 34483916 | 34660804 | NO CNV |  | NO |
| 8 | 8q21.2 | 255 |  | 4;2;1 |  | 0;0;1 | RP11-509F16 RP11-694L21 | 86620595 | 86875933 | Locus 2602 | ROXO1L1 | NO |
| 8 | 8q12.1 | 152 | 3 | 5 |  | 6 | RP11-513O17 | 58226061 | 58378511 | Locus 2547 |  |  |
| 8 | 8q24.23 | 186 |  | 1 |  | 1 | RP11-356M23 | 137793540 | 137979653 | Locus 2689 |  |  |
| 9 | 9p13.1 | 152 | 1 | 1 |  |  | RP11-293C6 | 39149715 | 39302027 | Locus 2780 | CNTNAP3 |  |
| 10 | 10q11.22 | 744 | 1;3;1;1;1 |  | 0;2;0;0;0 | 0;1;0;0;0 | RP11-314P12 RP11-292F22 RP11-192A16 RP11-30N1 RP11-115A11 | 46487806 | 47231326 | Locus 2984 | PPYR1, ANXA8L1, ANXA8 | YES |
| 10 | 10q23.2 | 177 |  | 2 |  |  | RP11-322M19 | 88946983 | 89123875 | Locus 3048 | FAM22A, FAM22D |  |
| 14 | 14q11.2 | 196 | 4 | 2 | 2 | 7 | RP11-449I24 | 19289488 | 19485285 | Locus 3738 | OR4M1, OR4N2, OR4K2, OR4K5, OR4K1 |  |
| 15 | 15q11.2 | 609 | 4;3;4;4;4 | 5;2;2;1;2 | 2;1;1;2;0 | 2;1;1;0;0 | RP11-118E23 RP11-717D19 RP11-32B5 RP11-2F9 RP11-603B24 | 19484741 | 20094181 | Locus 3879 | OR4M2,OR4N4,LOC650137 |  |
| 15 | 15q25.3 | 172 | 1 | 2 |  |  | RP11-103E2 | 84687961 | 84859560 | NO CNV | AGBL1 | NO |
| 16 | 16p11.2 | 2627 | 1;2;0;1;0 | 3;3;1;0;1 | 0;0;0;2;1 | 0;2;0;0;1 | RP11-545B4 RP11-264M14 RP11-274A17 RP11-577D6 RP11-488I20 | 31864841 | 34491828 | Locus 4060 | LOC729355, TP53TG3 |  |
| 16 | 16q22.1 | 201 | 2 | 3 | 6 | 3 | RP11-530B22 | 68598107 | 68799077 | Locus 4104 | PDPR, MGC34761 |  |
| 19 | 19q13.42 | 686 | 1;1 |  | 1;0 | 1;0 | CTD-2337J16 RP11-749K3 | 59383665 | 60069408 | Locus 4536 | MB0AT7, TSEN34, RP59, LILRB3, LILRA6, LILRB5, LILRB4, KIR3DL3, KIR2DL3, KIR2DL1, KIR2DL4, KIR2DS4, KIR3DL2 |  |
| 20 | 20p12.3 | 16 | 1 |  |  |  | RP5-1140M3 | 8198435 | 8214296 | Locus 4567 | PCLB1 |  |
| 22 | 22q11.1 | 114 | 1 |  |  |  | CTD-2177M20 | 14440103 | 14554197 | Locus 4746 |  |  |
| 22 | 22q11.1 | 499 | 2;1;1 | 1;0;0 |  |  | RP11-377E20 RP11-532J16 RP11-561P7 | 14453375 | 14952669 | Locus 4746 | A26C3, OR11H1 |  |
| 22 | 22q11.1 | 209 | 2 | 2 |  |  | RP11-803J7 | 16096956 | 16305835 | Locus 4746 |  |  |
| 22 | 22q11.21 | 88 | 1 | 2 |  |  | CTD-2280L11 | 18945707 | 19033820 | Locus 4746 |  |  |
| 22 | 22q11.21 | 159 | 1 | 3;1 |  |  | RP11-818K20 RP11-444L7 | 19847992 | 20006849 | Locus 4746 |  | YES (V24) YES (V8) |
| 22 | 22q11.21 | 18050 | 1 | 2 |  |  | CTD-2295P14 | 20105069 | 20155079 | Locus 4746 | HIC2 |  |
| 22 | 22q11.23 | 76 | 1 |  |  |  | CTD-2300P17 | 23233656 | 23310014 | NO CNV | UPB1, C22orf13, SNRPD3 | NO |
| 22 | 22q11.23 | 258 | 1;1 | 1;1 |  | 0;1 | RP11-50I7 RP11-261O10 | 23941648 | 24199456 | Locus 4753 | CRYBB2, IGLL3, LRP5L |  |
| 22 | 22q13.2 | 142 | 1 |  |  |  | RP11-138G4 | 39422672 | 39564831 | NO CNV | SLC25A17, ST13 | YES |
| X | Xq22.2 | 233 | 1 |  | 1 |  | RP11-402H1 | 102974612 | 103207477 | Locus 4914 | MGC39900, H2BFWT |  |
| Y | Yq11.223 | 4325 | 1;1;1;1;1;1;1;1;1;3 |  | 0;0;1;0;1;1;0;1;0;0 |  | RP11-241N3 RP11-376E17 CTD-2532F12 RP11-823D8 RP11-506M9 RP11-214M24 RP11-100J21 RP11-245K4 RP11-270H4 RP11-13O24 | 22402126 | 26727178 | Locus 4981 | PRY, RBMY1J, RBMY1E, PRY2, RBMY1B, RBMY1F, RBMY1A1, RBMY1D, PRY, DAZ1, DAZ4, BPY2C, PRY2, DAZ2, BPY2B, DAZ3, BPY2, CDY1B, CDY1 |  |

**Supplementary Table S2**

| **Chr Band** | **Target Clone** | **MLPA probe** | **START probe** | **END probe** | **Upstream hybridising sequence** | **Downstream hybridising sequence** |
| --- | --- | --- | --- | --- | --- | --- |
| 1p21.3 | RP11-29O12 | RP11-29O12 | 96593985 | 96594033 | gagctgcacccacatgtgccta | ttcagcgccaggatgctgttttactgc |
| 1p21.3 | RP11-29O12 | RP11-29O12b | 96488449 | 96488504 | gggccagaagtggtatatgacatggt | tgagacaattgatgacctttctccctattt |
| 1q21.1 | RP11-315I20 | GNRH2 | 144226590 | 144226642 | gaagtttccttacacctgaacgggatc | caagcgggttgagtactgctgcctgg |
| 1q21.1 | RP11-293J20 | PIAS3 | 144291617 | 144291679 | gctcgactctcagccactgttcccaac | accattgtggtcaattggtcatctgagttcggacgg |
| 1q21.1 | RP11-533N14 | BCL9 | 145481202 | 145481277 | ctgatatgagccccgcattctaagtccgaatacat | aggcccaacctctgtgctatctgg |
| 1q21.1 | RP11-301M17 | NBPF1 | 146293510 | 146293542 | cggaacactttggatgcggcttatggtgggtct | cggaacactttggatgcggcttatggtgggtct |
| 5q35.1 | RP11-292M11 | RP11-292M11 | 169642831 | 169642890 | gaaagatcccaccccagcccattcgca | atgtgttgataattctcccgttcacac |
| 5q35.1 | RP11-292M11 | RP11-292M11b | 169744026 | 169744084 | gatggtgcttcaccagattaagagtccagcctgga | atcgcttcagcgttgtgaagagtg |
| 8p12 | RP1-144M5 | RP1-144M5 | 34531103 | 34531158 | cagataatcagggctccctcttttctt | tccaaagggagaacaggggcatggccaag |
| 8p12 | RP1-144M5b | RP1-144M5b | 34641596 | 34641651 | gtgtgtggattggggggtctaaa | tatcatcccacacactagcagactagttaagtg |
| 8q21.2 | RP11-509F16 | RP11-509F16 | 86673078 | 86673136 | gtggacaatgcttaaagaaagcagagcct | tgtgtaattacctgaatgcacagtctattg |
| 8q21.2 | RP11-509F16b | RP11-509F16b | 86632867 | 86632927 | cagtaactcattgcctcagttgctctat | aagacatagaagaaaagctgtttgggataagag |
| 10q11.22 | RP11-314P12 | PPYR1 | 46507740 | 46507809 | caacccattcatctatggctttctcaacacc | aacttcaag aaggagatcaaggccctggtgctgacttgc |
| 15q25.3 | RP11-103E2 | RP11-103E2 | 84743979 | 84744038 | cacattatttccagctcacagctggttgctttggat | tcccaaatggcaggccagcttctc |
| 15q25.3 | RP11-103E2b | RP11-103E2b | 84843618 | 84843673 | gggttggggtctgtggctatgcaa | tttcgaacgaagtgtaatggaaccagg |
| 22q11.21 | RP11-444L7 | RP11-444L7 | 19995363 | 19995422 | atgtataagccctaaagcaacgagcccaag | aggccccagacactgcccatcatcataaag |
| 22q11.23 | CTD-2300P17 | C22orf13 | 23267064 | 23267120 | gaccagtctagagccaacaccggtctc | tgaaacccagaagggctgcccaactaacag |
| 22q11.23 | CTD-2300P17 | UPB1 | 23251796 | 23251844 | cacctctgccccagtggattagca | agtgtggcaggcttaacatgtccag |
| 22q13.2 | RP11-138G4 | SLC25A17b | 39499838 | 39499893 | gatggctgctgaaggagagcacggaatg | tgaggcatctacaaagctcaccttactc |

**Supplementary Table S3**

| **Chr Band** | **Target Gene** | **START probe** | **END probe** | **Upstream hybridising sequence** | **Downstream hybridising sequence** |
| --- | --- | --- | --- | --- | --- |
| 22q11.2 | HIRA | 17698971 | 17699021 | ccctcaggatgtcgagctgttcctgac | actcggtgaagaggcgctggaatc |
| 1p13.2 | NGFB | 115635119 | 115635174 | ctaccctgccttgcatctgctggtttga | ctgaactgacttcacaaagttcctacac |
| 1p36.33 | GABRD | 1949185 | 1949243 | catggcacggattgatattgttgagccagtgc | agcagcccctgtgtgtcacctgacaac |
| **1q21.1** | **PIAS3** | **144291617** | **144291679** | gctcgactctcagccactgttcccaac | accattgtggtcaattggtcatctgagttcggacgg |
| 4p16.1 | DRD5 | 9552719 | 9552778 | caacggcagggaccacaatcactccaaaca | ggtatggccaggtactctcctgtgggagaa |
| 22q11.2 | COMT | 18335516 | 18335575 | ctggtgaagatggggggtctgcaaatgcag | gagcttggggatgtccagaactgaccccaa |
| **1q21.1** | **BCL9** | **145481202** | **145481277** | ctgatatgagccccgcattctaagtccgaatacat | aggcccaacctctgtgctatctgg |
| **1q21.1** | **NBPF1** | **146293501** | **146293577** | cggaacactttggatgcggcttatggtgggtct | cggaacactttggatgcggcttatggtgggtct |
| 6p22.1 | GABBR1 | 29681943 | 29682003 | gccctaagctcctcatagcaaaagagcaac | tctcccctattctcagaaaagattagtgca |
| 3q26.3 | NLGN1 | 174791897 | 174791956 | caggaaaccattattgctgttcggtgcctt | atgtgaaaggcgttggtggtcttagtccag |
| 1p36.11 | SMP1 | 25539567 | 25539658 | gtctggatttctagagggcttgagatgctcagaatgcattgactgggggga | aaagcgcaatactattgcttccattgctgctggtgtactagt |
