# Supplementary figures and images for: BAC array CGH in patients with Velocardiofacial syndrome-like features reveals genomic aberrations on chromosome region 1q21.1
Source: BMC Med Genet. 2009 Dec 23;10:144. doi: 10.1186/1471-2350-10-144 (PMC2805625; doi:10.1186/1471-2350-10-144)

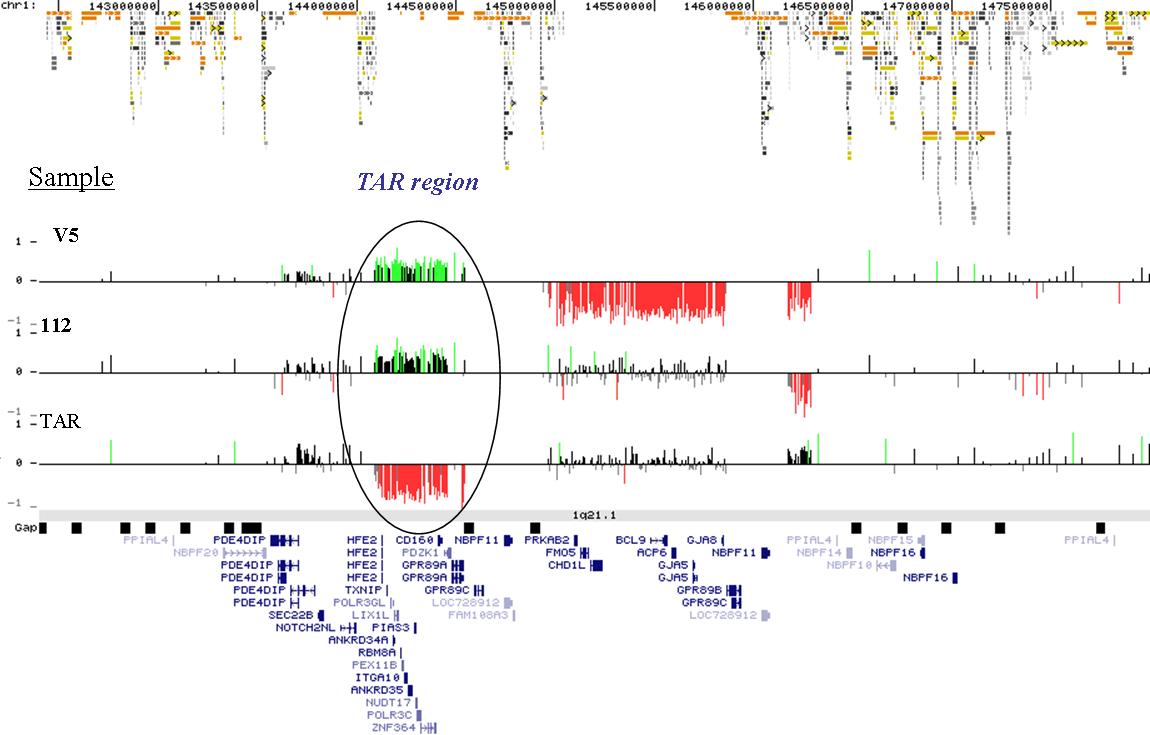

Supplement: Additional file 2 — Supplementary Figure S4. Array CGH results for deletion cases V5 and 112 (Build 36, chr1:143,500,000-148,000,000). For each patient, deviations from 0 of probe log2 ratios are depicted by vertical bars, with those exceeding a threshold of 1.5 SD from the mean probe ratio shown in green (gains) or red (losses). The region of the microdeletion associated with TAR syndrome is indicated with a circle. [file 1471-2350-10-144-S2.JPEG]
